# Comparison of different approaches for direct coupling of solid-phase microextraction to mass spectrometry for drugs of abuse analysis in plasma

**DOI:** 10.1016/j.jpha.2022.10.004

**Published:** 2022-11-09

**Authors:** Wei Zhou, Martyna N. Wieczorek, Runshan Will Jiang, Janusz Pawliszyn

**Affiliations:** a Department of Chemistry, University of Waterloo, Waterloo, ON, N2L 3G1, Canada; b Faculty of Food Science and Nutrition, Poznań University of Life Sciences, Poznań, Poland

**Keywords:** Solid-phase microextraction, Mass spectrometry, Microfluidic open interface, Coated blade spray, Probe electrospray ionization, Drug of abuse

## Abstract

The direct coupling of solid-phase microextraction (SPME) to mass spectrometry (MS) (SPME-MS) has proven to be an effective method for the fast screening and quantitative analysis of compounds in complex matrices such as blood and plasma. In recent years, our lab has developed three novel SPME-MS techniques: SPME-microfluidic open interface-MS (SPME-MOI-MS), coated blade spray-MS (CBS-MS), and SPME-probe electrospray ionization-MS (SPME-PESI-MS). The fast and high-throughput nature of these SPME-MS technologies makes them attractive options for point-of-care analysis and anti-doping testing. However, all these three techniques utilize different SPME geometries and were tested with different MS instruments. Lack of comparative data makes it difficult to determine which of these methodologies is the best option for any given application. This work fills this gap by making a comprehensive comparison of these three technologies with different SPME devices including SPME fibers, CBS blades, and SPME-PESI probes and SPME-liquid chromatography-MS (SPME-LC-MS) for the analysis of drugs of abuse using the same MS instrument. Furthermore, for the first time, we developed different desorption chambers for MOI-MS for coupling with SPME fibers, CBS blades, and SPME-PESI probes, thus illustrating the universality of this approach. In total, eight analytical methods were developed, with the experimental data showing that all the SPME-based methods provided good analytical performance with *R*^2^ of linearities larger than 0.9925, accuracies between 81% and 118%, and good precision with an RSD% ≤ 13%.

## Highlights

• Rapid screening and quantitative analysis were achieved by SPME-MS direct coupling.
• SPME with different geometries including fiber, blade and probe were studied.
• Three SPME-MS techniques including MOI-MS, CBS-MS and PESI-MS has been investigated.
• High throughput SPME were achieved by automated 96 Concept system.
• The methods showed good analytical performance for drugs of abuse analysis.

## Graphical abstract

**Figure:**
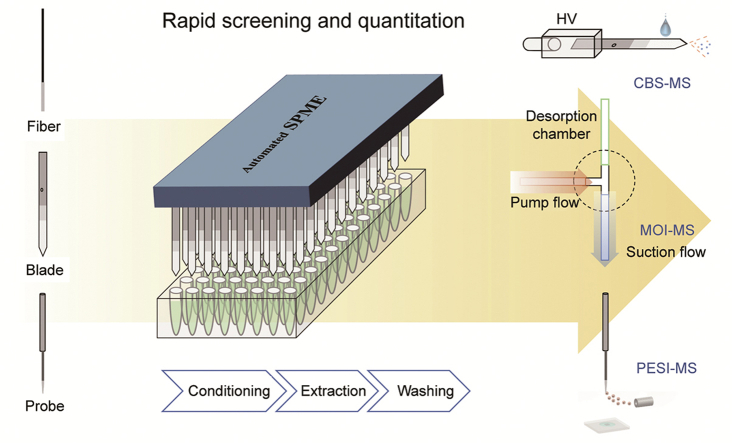

## 1 Introduction

Mass spectrometry (MS) is a powerful and popular tool that provides not only highly specific molecular information, including molecular weights and chemical structures, but also sensitive and selective quantitative analysis with fast scanning speeds. Normally, MS is coupled with chromatographic separation techniques like gas chromatography (GC), liquid chromatography (LC), capillary electrophoresis (CE), and capillary electrochromatography (CEC) [[1], [2], [3], [4]]. However, chromatography-based separation methods require tedious sample preparation and long separation time, making them both time and labor intensive. The highly selective detection afforded by the development of tandem MS, high-resolution MS, ion mobility-MS, and direct and ambient MS techniques has enabled the removal of the chromatographic separation process from the workflow, thereby shortening turnaround time and enabling rapid screening. Recently, direct and/or ambient MS techniques such as desorption electrospray ionization (DESI) and direct analysis in real time (DART) have become major focal points of research in many fields [5,6]. Direct/ambient MS is ideal for clinical point-of-care and anti-doping analysis, which not only requires highly specific analytical results but also fast screening or even on-site capabilities for diagnosing diseases or informing therapeutic regimens. For example, coupling paper extraction nanoelectrospray with portable MS has already been studied for the fast determination of 2-hydroxyglutarate in human brain samples to determine its viability as a biomarker for distinguishing isocitrate dehydrogenase (IDH) I and II mutations in gliomas [7]. Unfortunately, working with complex matrices such as biofluids and tissues can be problematic, as interferences and ion suppression from these matrices can result in MS contamination and reduced sensitivity [8,9]. As such, frequent maintenance of the MS instrument is required to avoid contamination [10].

Solid-phase microextraction (SPME) is a simple yet highly effective sample-preparation technique that consolidates sampling, extraction, clean up, and enrichment into a single step [11,12]. The direct coupling of SPME and MS has been proven to be an effective method of addressing the aforementioned issues related to complex matrices [13]. In particular, biocompatible SPME (bio-SPME) coatings are especially effective in minimizing matrix effects when working with biological samples, as they feature solid particles embedded into a polymeric binder (e.g., polyacrylonitrile (PAN)), which enables the exclusion of large cells and proteins [14]. Given these advantages, researchers in many areas have sought to couple various SPME geometries (e.g., fibers, needles, blades, and meshes) with different kinds of interfaces, such as nano-electrospray ionization (nano-ESI), microfluidic open interface (MOI), and probe electrospray ionization (PESI) [[15], [16], [17]].

Among all the SPME-MS direct coupling techniques, MOI-MS is the only concept to contain a flow-isolated desorption chamber that allows the desorption of SPME devices with small amounts of solvents (less than 10 μL) [18]. The interface provides an open-to-ambient desorption system, which allows SPME devices to be desorbed using small amounts of solvent, followed by direct injection into the MS for fast analysis. Coated blade spray-mass spectrometry (CBS-MS) is another SPME-MS direct coupling developed by our lab that has been commercialized in recent years [[19], [20], [21]]. The analytes are extracted and enriched in the coating and desorption takes place directly on the surface of the blade. Finally, the analytes are introduced to the MS by applying high voltage to the metal blade via substrate-based electrospray ionization (ESI). In SPME-PESI, extraction is performed using a small, sharp metal SPME fiber thinly coated with a solid sorbent, with desorption and ionization being achieved using a repetitive pick-and-spray cycle with pL levels of desorption solvent [17]. The three above-discussed SPME-MS direct coupling techniques have different features and are suitable for different applications, but the lack of a comprehensive comparison of these methods makes it difficult to select the best option from lab to lab, or between instrumental companies and industries. Moreover, the use of different MS instruments for these interfaces can make the developed methodologies confusing for users, as there is significant variance in the performance of MS instruments, including their sensitivity, repeatability, scan speed, and set up.

In this work, we conducted a comprehensive comparison of the SPME-MOI-MS, CBS-MS, SPME-PESI-MS, and SPME-LC-MS protocols, using different SPME devices to analyze drugs of abuse with Log P values ranging from 1.2 to 4.1 from plasma samples, followed by analysis with the same Shimadzu 8060 MS (Kyoto, Japan), as this instrument offers good sensitivity and fast scan speeds. In addition, a novel MOI-MS interface with different shapes of desorption chambers was designed for coupling with SPME devices with different geometries. Eight different analytical methods were developed with respect to their sensitivity, repeatability, accuracy, and precision. The analytical results from the comparison of different SPME-MS interfaces with the same instrument will be helpful in further development and application of the examined techniques.

## 2 Experimental

### 2.1 Chemicals and instrumentation

MS-grade solvents, including methanol (MeOH), acetonitrile (ACN), isopropanol (IPA), and water, were purchased from Fisher Scientific (Hampton, NJ, USA), while formic acid (FA) was supplied by Sigma Aldrich (Oakville, ON, Canada). 1 × phosphate buffer saline (PBS) solution (pH 7.4) was prepared by dissolving PBS solid particles obtained from Sigma Aldrich into water. Drug-of-abuse standards, including codeine, diazepam, fentanyl, cocaine, nordiazepam, oxazepam, and propranolol, and their isolable internal standards (ISs), including codeine-d_3_, diazepam-d_5_, fentanyl-d_5_, cocaine-d_3_, nordiazepam-d_5_, oxazepam-d_5_, and propranolol-d_7_ were purchased from Cerilliant (Round Rock, TX, USA). All standards and ISs were dissolved in MeOH or ACN at a concentration of 1 mg/mL (standards) or 0.1 mg/mL (ISs). Finally, the human plasma (pooled gender) used in this work was purchased from Bioreclamation IVT (Westbury, New York, USA).

MS analyses were performed on a Shimadzu MS 8060 triple quadrupole mass spectrometer equipped with a Shimadzu LC-30AD system, an ESI interface, and a Shimadzu DPiMS-8060 PESI interface. The heater cable assy was generously provided by Shimadzu (Columbia, MD, USA). After connecting the cable to the front panel of the MS, the instrument can be run without the commercial ESI or PESI interface. For detection, the dwell time was set at 10 ms for each multiple reaction monitoring (MRM) transition, with two MRM transitions being selected for each compound, one for quantitative analysis and the other one for qualitative analysis. Information related to the MRM transitions is provided in Table S1. CBS blades with hydrophilic-lipophilic balanced (HLB) particles (5 μm diameter) embedded in PAN were generously provided by Restek (Bellefonte, PA, USA). The coating on the CBS blade was 15 μm thick on each side, and 1 cm in length. SPME fibers were coated with HLB particles (5 μm diameter) in the lab to a thickness of 20 μm and a length of 1 cm using the dip-coating method. SPME-PESI probes were coated with home-made HLB particles (1.3 μm diameter) to a thickness of 6.5 μm and a length of 2 mm according to the method described in a previous study [17]. It should be emphasized that because the PESI probe is very thin, HLB particles with small diameters should be used to ensure a thin and uniform coating. A Concept 96 system (PAS Technology, Magdala, Germany) was integrated into the fiber- and blade-based SPME protocols to facilitate the automation of the preconditioning, extraction, washing, and desorption (desorption only for LC-MS) steps, as well as high-throughput analysis by enabling the use of up to 96 fibers or blades simultaneously. The holders for the SPME fibers and blades were fabricated by the University of Waterloo's Science Technical Services, while the holder for the SPME-PESI probe was fabricated using a 3D printer. The details of this process are discussed in greater depth in Section 3.

For LC-MS, a Phenomenex (Torrance, CA, USA) Kinetex PFP column (2.1 mm × 100 mm) with 1.7 μm particle was used. The flow rate was 300 μL/min. The mobile phase A was water with 0.1% formic acid and mobile phase B was ACN with 0.1% formic acid. The gradient was 10% B for 1.0 min, then linearly ramped to 100% B until 7.0 min, held there until 9.0 min, and then returned to 10% B at 9.2 min. This composition was kept until 11.0 min. The column oven temperature was set as 40 °C. Sample injection volume was 5 μL. For MS, the interface voltage was 4.0 kV in positive mode, the interface temperature was 300 °C, the desolvation line temperature was 250 °C, the heating block temperature was 400 °C, and the nebulizing gas, drying gas and heating gas flow were 3.0, 10.0 and 10.0 L/min, respectively. The collision gas pressure was 270 kPa with argon. For MOI-MS, the interface temperature was decreased to 100 °C to avoid the boiling of desorption solvents. Other MS parameters were the same as those of LC-MS. For CBS-MS, the interface was opened, and the interface heating as well as the nebulizing gas, drying gas and heating gas were closed. The other MS parameters were the same as the above-mentioned ones. The distance between CBS tip and the MS inlet was 1 cm. For PESI-MS, the ESI voltage was 2.3 kV in positive mode, heating block temperature was 30 °C, pause time was 1 ms, and dwell time was 1 ms.

### 2.2 Analytical protocols

**Fig. 1:**
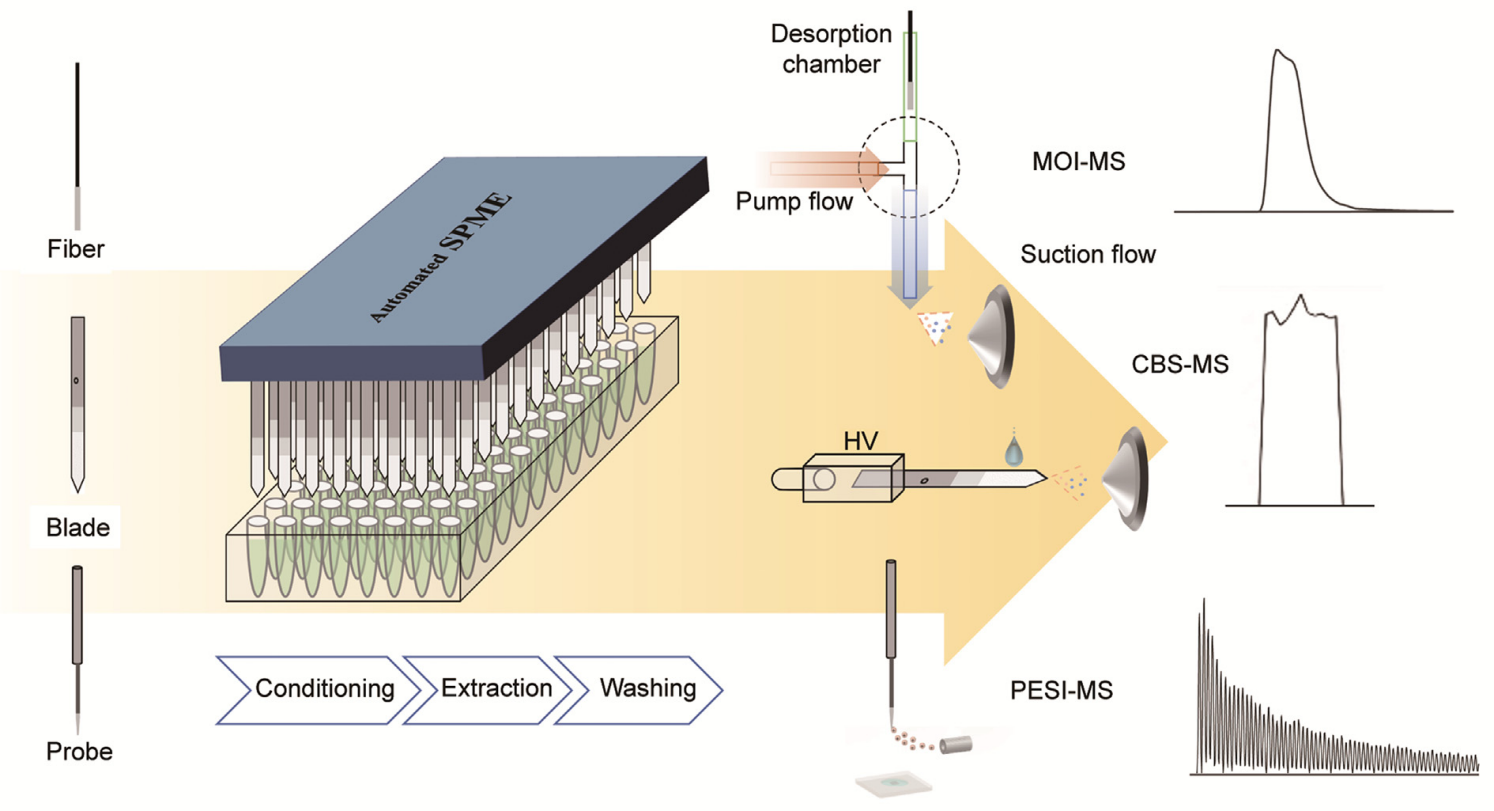
Schematic of solid-phase microextraction (SPME) devices, SPME processes, solid-phase microextraction-mass spectrometry (SPME-MS) direct coupling devices, and typical MS spectrums. HV: high voltage; MOI-MS: microfluidic open interface-mass spectrometry; CBS-MS: coated blade spray-mass spectrometry; PESI-MS: probe electrospray ionization-mass spectrometry.

To construct the calibration curves and validate the methods, the standards were spiked into 300 μL plasma at concentrations of 0.05, 0.1, 0.25, 1, 10, 25, 50, and 100 ng/mL, while internal standards were spiked at a concentration of 10 ng/mL, with the exception of fentanyl (5 ng/mL) and cocaine (1 ng/mL). All plasma samples were stored overnight in a refrigerator at 4 °C prior to use to allow drug-protein binding equilibrium to be achieved. Before extraction, all plasma samples were diluted with 900 μL of PBS solution. A schematic diagram of the complete SPME-MS direct coupling protocol is presented in Fig. 1, and a photograph of the Concept 96 system is provided in Fig. S1. Without specific declaration, 1.2 mL of sample (i.e., 300 μL of plasma diluted with 900 μL of PBS solution) was added to a 2 mL 96-well plate for extraction. The SPME fibers or blades were then inserted into the Concept 96 fiber or blade holder, preconditioned in an MeOH/H_2_O solution for 20 min, and immersed in the sample for 20 min with agitation at 1500 rpm. After the 20 min extraction period, the fibers or blades were washed in H_2_O for 5 s. Next, the SPME devices were desorbed in the Concept 96 system for 30 min in 300 μL of a solution consisting of MeOH/ACN/H_2_O (7:2:1, *V/V/V*) + 0.1% FA, with the solutions subsequently being transferred to LC vials with insert tube for LC-MS analysis. For the direct MS analysis, desorption and injection were conducted immediately prior to direct MS analysis. The PESI probes were desorbed for LC-MS analysis in LC vials with insert tube for 30 min in 100 μL of desorption solution. Based on our previous work, the following desorption conditions were applied: for CBS-MS, 8 μL of MeOH/H_2_O (95:5, *V/V*) + 0.1% FA was applied to the blade for 18 s; for SPME-MOI-MS, the fiber was immersed in MeOH/ACN/H_2_O (7:2:1, *V/V/V*) + 0.1% FA in the desorption chamber for 10 s; and for SPME-PESI-MS, pL levels of an IPA/H_2_O (5:5, *V/V*) + 0.1% FA solution were applied via a repetitive pick-and-spray cycle for 30 s. Each calibration and validation point was tested in quadruplicate. A total of eight calibration curves were constructed (i.e., SPME fiber-LC-MS, SPME blade-LC-MS, SPME-PESI probe-LC-MS, SPME fiber-MOI-MS, SPME blade-MOI-MS, SPME-PESI probe-MOI-MS, CBS-MS, and SPME-PESI-MS), with each curve containing three validation points.

## 3 Results and discussion

### 3.1 Design of the devices and general description of three SPME-MS techniques

As shown in Fig. S1, the Concept 96 system allows 96 samples and devices to be employed at the same time and all four agitators to be set to different agitation speeds and time, with the holders containing the SPME devices being moved by the auto arm [22].

The MOI-MS interface is based on the concept of a flow-isolated desorption chamber. In the ESI-MS interface, the nebulizer gas causes the Venturi effect, which drives a constant suction flow rate in the ESI pipeline [18]. One port of the three-port tee was connected to the inlet of the ESI needle, the second port was connected to the syringe pump, which was used to deliver the solvent, and the third port was connected to the desorption chamber. Fundamentally, the shape of the desorption chamber can be designed to accommodate different SPME devices. In this work, two new desorption chamber designs capable of accommodating SPME blades and small SPME-PESI probes were introduced for the first time and applied for SPME blade-MOI-MS and SPME-PESI probe-MOI-MS. Photos of these three chambers and the MOI-MS interfaces are shown in Fig. S2.

CBS-MS interface is very simple, consisting of an *x–y–z* stage for precisely positioning the CBS blade in front of the MS inlet, a holder for inserting the CBS blade, and an electronic contact inside the holder for applying high voltage. For desorption, 8 μL of desorption solution was applied to the blade coating, with desorption occurring on the coating. After 18 s, high voltage was applied to the stainless-steel blade, spraying and ionizing the desorption solution containing the analytes. There is no need for nebulizer gas or high temperatures in CBS-MS, as the spraying takes place in ambient conditions, which allows it to be easily coupled with portable MS for on-site analysis.

Commercial PESI-MS uses a repetitive pick-and-spray cycle in which the PESI probe picks up pL levels of sample/solvent in each cycle, followed by the application of high voltage to induce spraying in front of the MS. This cycle is repeated for a pre-designated number of times to obtain a reproducible MS signal. In SPME-PESI-MS, the target analytes were concentrated on the coating, and desorption solution was picked up every cycle for desorbing the analytes and inducing ionization. Every spray cycle was 250 ms and the total analytical time was 30 s; all peak areas during these sprays were integrated.

### 3.2 Comparison of the eight different analytical methods for the analysis of plasma samples

A comprehensive comparison of the SPME-LC-MS (×3), SPME-MOI-MS (×3), CBS-MS, and SPME-PESI-MS methods was made by applying them for the quantitative analysis of seven drugs of abuse in the plasma samples, as the anti-doping tests must be able to provide fast on-site analysis during competitions. The typical MS spectrums for three SPME-MS methods in the analysis of seven drugs of abuse in plasma are shown in Fig. S3. The influence of extraction time and sample volume is shown in Fig. S4, but for comparison, the same extraction time and sample volume as indicated in experimental section were used for all the methods. The analytical data including slope, intercept, *R*^2^, limits of detection (LOD), and limits of quantitation (LOQ) are shown in Table 1, Table 2. The LODs were estimated based on the presence of a signal-to-noise ratio (S/N) larger than 3 compared with blank matrix extraction, and the LOQs were defined by calibration or validation points where the S/N was larger than 10 and the precision (relative standard deviation (RSD%) of four replicates) was less than 20%, as well as the linearity should pass the lack-of-fit test. The lack-of-fit test was used for assessment of the linearity. If the F (test) is less than the F (critical), it means that the linearity has 95% confidence level. As shown in Tables S2 and S3, except the SPME fiber-MOI-MS method for oxazepam analysis, all the other calibration curves had 95% confidence level for linearity. For SPME-PESI-MS and SPME-PESI probe-LC-MS, there were no calibration curves available for some of the compounds because the spiked concentrations of the related internal standards were below the LOQs, so there was no data for peak area of analyte/internal standard available. As this study aimed for the comparison of different methods, we decided to keep all the spiking amount to be the same, to decrease the experiment error and to make them more comparable. But it should be emphasized that in real application using SPME-PESI-MS, the spiking amount of ISs should meet the LOQs of the method for quantitative analysis.

**Table 1:** Quantitative results of drugs of abuse analysis in plasma using different solid-phase microextraction (SPME)-mass spectrometry (MS) direct coupling methods.

Compounds	SPME fiber-MOI-MS					CBS blade-MOI-MS					SPME-PESI probe-MOI-MS					CBS-MS				
	Slope	Intercept	*R*^2^	LOD (ng/mL)	LOQ (ng/mL)	Slope	Intercept	*R*^2^	LOD (ng/mL)	LOQ (ng/mL)	Slope	Intercept	*R*^2^	LOD (ng/mL)	LOQ (ng/mL)	Slope	Intercept	*R*^2^	LOD (ng/mL)	LOQ (ng/mL)
Codeine	0.0953	0.0651	0.9998	1.00	3.00	0.0653	0.1708	0.9972	1.00	3.00	/	/	/	10.00	25.00	0.2170	0.4596	0.9989	1.00	3.00
Diazepam	0.1243	−0.1223	0.9997	1.00	3.00	0.1784	−0.2839	0.9985	0.50	1.00	/	/	/	10.00	25.00	0.0505	−0.1214	0.9985	0.25	3.00
Fentanyl	0.1747	−0.0006	0.9996	0.01	0.05	0.1641	0.0559	0.9991	0.01	0.05	0.1890	0.0471	0.9997	0.05	0.10	0.1949	−0.0113	0.9996	0.01	0.05
Cocaine	0.8777	−0.0602	0.9999	0.03	0.10	0.8645	0.1178	0.9995	0.02	0.05	/	/	/	3.00	10.00	0.9120	0.0570	0.9996	0.02	0.10
Nordiazepam	0.1558	−0.0337	0.9998	0.05	1.00	0.1545	0.0132	0.9997	0.10	0.25	0.1562	−0.0259	0.9998	3.00	10.00	0.1563	−0.0698	0.9997	0.10	1.00
Oxazepam	0.0931	−0.2486	0.9925	1.00	3.00	0.1202	0.2517	0.9994	1.00	3.00	/	/	/	10.00	25.00	0.0860	0.1417	0.9997	3.00	10.00
Propranolol	0.2439	−0.0620	0.9999	0.05	0.10	0.2339	0.0440	0.9995	0.05	0.10	0.2435	0.0600	0.9999	0.10	0.25	0.2169	−0.0720	0.9998	0.02	0.05

/: calibration curve no data available; MOI: microfluidic open interface; CBS: coated blade spray; PESI: probe electrospray ionization; LOD: limit of detection; LOQ: limit of quantitation; The calibration ranges for all methods were from LOQs to 100 ng/mL.

**Table 2:** Quantitative results of drugs of abuse analysis in plasma using solid-phase microextraction-probe electrospray ionization-mass spectrometry (SPME-PESI-MS) and SPME-liquid chromatography-mass spectrometry (SPME-LC-MS) with different devices.

Compounds	SPME-PESI-MS					SPME fiber-LC-MS					CBS blade-LC-MS					SPME-PESI probe-LC-MS				
	Slope	Intercept	*R*^2^	LOD (ng/mL)	LOQ (ng/mL)	Slope	Intercept	*R*^2^	LOD (ng/mL)	LOQ (ng/mL)	Slope	Intercept	*R*^2^	LOD (ng/mL)	LOQ (ng/mL)	Slope	Intercept	*R*^2^	LOD (ng/mL)	LOQ (ng/mL)
Codeine	0.0915	−0.1813	0.9992	3.00	10.00	0.0912	0.1324	0.9997	1.00	3.00	0.0963	−0.0753	0.9997	0.25	1.00	/	/	/	50.00	/
Diazepam	/	/	/	10.00	25.00	0.1022	0.2522	0.9995	1.00	3.00	0.1193	−0.1321	0.9996	1.00	3.00	/	/	/	25.00	75.00
Fentanyl	0.1781	−0.0967	0.9992	0.01	0.05	0.1860	0.0833	0.9996	0.05	0.10	0.1990	−0.0683	0.9997	0.02	0.05	0.1770	0.1514	1.0000	1.00	3.00
Cocaine	/	/	/	3.00	10.00	0.7364	0.9707	0.9993	0.05	1.00	0.8081	−0.1353	0.9999	0.05	0.10	/	/	/	3.00	10.00
Nordiazepam	0.2378	−1.3305	0.9930	3.00	10.00	0.1468	−0.0846	0.9959	1.00	3.00	0.1643	−0.0846	0.9998	0.25	1.00	/	/	/	10.00	30.00
Oxazepam	/	/	/	25.00	50.00	0.1065	0.1568	0.9997	1.00	3.00	0.1198	−0.0512	0.9999	0.25	1.00	/	/	/	25.00	75.00
Propranolol	0.2351	−0.3013	0.9992	0.25	1.00	0.2259	0.0910	0.9999	0.25	1.00	0.2466	−0.3352	0.9999	0.10	3.00	0.2169	0.1562	0.9998	3.00	10.00

/: calibration curve no data available; CBS: coated blade spray; LC-MS: liquid chromatography-mass spectrometry; LOD: limit of detection; LOQ: limit of quantitation; The calibration ranges for all methods were from LOQs to 100 ng/mL.

As indicated by the results in Table 1, all the three SPME-MOI-MS direct coupling methods showed good linearity, with an *R*^2^ ≥ 0.9925, with the CBS blade-MOI-MS method showing the best sensitivity, with the LODs of less than 1 ng/mL for most analytes. This result was due to the fact that the larger volume of extraction phase on the CBS blades enabled the extraction of greater amounts of analyte. In addition to improving its extraction kinetics, the large specific surface area of CBS blades also enhanced its desorption kinetics. The data acquired for the three different SPME-MS direct coupling methods are shown in Table 1, Table 2. As can be seen, all the three methods showed good linearity (*R*^2^ ≥ 0.9925), with the SPME fiber-MOI-MS and CBS-MS protocols showing similar sensitivity, although the CBS-MS protocol was slightly more sensitive. The data for the three SPME devices coupled with LC-MS after desorption are shown in Table 2. The CBS blade-LC-MS protocol clearly provided the best sensitivity for most of the compounds due to the largest extraction phase volume and specific surface area of the three devices. Interestingly, the data indicates that with the exception of codeine and oxazepam, the SPME-MS direct coupling methods provided better sensitivity for most of the compounds compared to the corresponding LC-MS methods. This result is because when using LC vials with an insert tube or a 96-well plate, a larger volume of desorption solvent is typically required to ensure the full immersion of the device and reproducible sample injection with the commercial LC-MS sample-injection system. However, for SPME-MS direct coupling, MS analysis takes place right after desorption with the small amounts of desorption solvent required by these methods, resulting in a high enrichment factor and better sensitivity. The validation data for three concentration levels (i.e., low, middle, and high) are shown in Tables S4–S6. As can be seen, all the eight SPME methods showed very good accuracy, with recoveries between 81% and 118%, and good precision with an RSD% for four replicates of ≤13%.

### 3.3 General comparison of three SPME-MS and SPME-LC-MS techniques

For routine LC-MS analysis, the LC run typically takes more than 10 min. In contrast, direct/ambient MS analysis provides fast analysis within 1 min, which is a highly appealing feature. In addition, the use of a high-throughput SPME system allows the automated analysis of 96 samples simultaneously; the totally sample preparation time for each sample is 25 s. For all the three direct SPME-MS coupling methods, desorption and MS quantification took about 30 s. Therefore, the total turn-around time for each sample was about 1 min. This is significantly faster than normal analytical methods, which involve tedious liquid extraction and chromatographic separation. Furthermore, SPME-MS direct coupling via CBS-MS and SPME-MOI-MS is simple and portable, and does not require the use of high-pressure pumps, which is very economical and potentially applicable for on-site analysis when combined with portable MS. Matrix effects have always been a major issue for direct/ambient-MS analysis methods such as paper spray and DESI-MS. According to our previous work, bio-SPME enables the elimination or significant reduction of both absolute and relative matrix effects through its cleaning step [23,24]. Sensitivity is another important factor. Although the desorption efficiency of directly coupled SPME-MS is lower than that of SPME-LC-MS methods, the smaller volumes of desorption solution allow SPME-MS to achieve higher enrichment factors and thus greater sensitivity. Of course, without LC separation, some matrix interferences with the same MRM transitions as the target compounds may cause high background noise, which decreases the LODs achieved with these methods. Nonetheless, this issue can be addressed by selecting more specific MRM transitions using high-resolution MS or ion mobility MS.

Comparing the three SPME-MS direct coupling methods, the new SPME-MOI-MS design with a simple three-port tee can be conveniently coupled with the commercial ESI or atmospheric pressure chemical ionization (APCI) interface by simply replacing the post-column pipeline from the LC, without requiring any modifications to the MS instrument. In addition, SPME devices with various geometries can be used with the MOI-MS interface by designing a desorption chamber with a compatible shape. Unlike CBS-MS, MOI-MS ionization takes place in a closed environment with controlled pressure and temperature. This allows the co-axial sheath gas and the heating gas to be used to enhance evaporation and ionization, which is crucial for analytes suffering from ionization efficiency in ambient conditions. Furthermore, the MOI-MS interface is also compatible with APCI-MS and electron ionization (EI)-MS interfaces, which is useful for the analysis of compounds that are more compatible with APCI or EI analysis. For CBS, the large extraction phase volume and high specific surface area of commercialized CBS blades enables high extraction capacity and fast extraction and desorption kinetics while using only a thin coating. For each spray, desorption and ionization only occur on one side of the blade, which means the two sides of the blade can be used for different purposes. For example, one side can be used for analysis in positive ionization mode and the other side can be used for analysis in negative ionization mode [21]. The CBS-MS protocol's most attractive feature is the simple design of its interface, making it a good candidate for fast on-site screening applications such as antidoping testing or point-of-care drug analysis. The spray time of CBS-MS can be extended to 20 s by adjusting the volume of desorption solution. If the MS instrument allows the dwell time to be set at 1 ms, the collection of the MS signal is 10 points for each MRM transition, with two MRM transitions being detected for each compound. In total, it is possible to monitor 2,000 MRM transitions and 1,000 compounds, which is beneficial for high-throughput screening [25]. We kept the sample volume at 1.2 mL for all tested SPME methods to ensure comparable experimental results; however, this approach was somewhat unfair to SPME-PESI-MS, as it can maintain fast extraction kinetics when using very small volumes of sample. Although SPME-PESI-MS had lower sensitivity for larger samples compared to SPME-MOI-MS and CBS-MS, mainly due to its use of a very small SPME probe with a small coating volume, the small probe size is advantageous when working with small volumes of sample (e.g., one drop of blood or even a single cell) due to its high enrichment factor [17]. SPME-PESI-MS can also be a very useful technique for investigating the free concentration of target analytes in a sample, as the small SPME-PESI probe offers negligible depletion of the free concentration of analytes in a sample, which does not significantly influence the analyte-matrix binding [26].

## 4 Conclusions and future perspectives

In this work, three recently developed SPME-MS direct coupling techniques using different geometries and SPME-LC-MS were studied using the same MS instrument. Several new designs were introduced, including MOI-MS desorption chambers specifically designed to accommodate SPME blades and SPME-PESI probes. Good analytical results were obtained for all the eight SPME methods, with *R*^2^ of linearities larger than 0.9925, accuracies between 81% and 118%, and good precision with RSD% ≤ 13%. The results of this comprehensive comparison can help researchers better understand SPME technologies and adopt suitable protocols for their particular research programmes. From the quantitative results and the purpose of rapid analysis, CBS-MS showed the best performance regarding the application in the analysis of drugs of abuse in plasma samples.

With respect to technique, future investigations can examine how these protocols can be improved through more precise design and total system automation. The full automation of an SPME-GC-MS system using a mechanical arm was achieved and commercialized many years ago and has since been demonstrated as a reliable technique that can be applied in different fields. There is very good potential to develop a fully automated SPME-MS direct coupling method, as the operation takes place in ambient and/or open-to-ambient systems. Regarding the applications of SPME-MS methods, although in-lab experimental results have already demonstrated their reliability for the fast screening and targeted quantitative analysis of drugs in bio-fluids like blood, plasma, urine, and saliva, more attention should be paid to the optimization of these techniques for on-site analysis, such as anti-doping testing during competitions and drug or biomarker monitoring in the surgery room during medical operations.

## CRediT author statement

**Wei Zhou:** Formal analysis, Investigation, Methodology, Validation, Visualization, Writing - Reviewing & Editing; **Martyna N. Wieczorek:** Methodology, Validation, Writing - Reviewing & Editing; **Runshan W. Jiang:** Methodology, Investigation; **Janusz Pawliszyn:** Conceptualization, Funding acquisition, Methodology, Supervision, Project administration, Writing - Reviewing & Editing.

## **Declaration of competing interest**

The authors declare that there are no conflicts of interest.
