# Supplementary material for: Comparison of different approaches for direct coupling of solid-phase microextraction to mass spectrometry for drugs of abuse analysis in plasma
Source: J Pharm Anal. 2022 Nov 9;13(2):216–22. doi: 10.1016/j.jpha.2022.10.004 (PMC9999297; doi:10.1016/j.jpha.2022.10.004)
Supplement: Multimedia component 1 [file mmc1.docx]

**Supplementary Material**

**Comprehensive comparison of different solid-phase microextraction devices and direct coupling interfaces to mass spectrometry**

Wei Zhou^a^, Martyna N. Wieczorek^a,b^, Runshan Will Jiang^a^, Janusz Pawliszyn^a*^

^a^Department of Chemistry, University of Waterloo, Waterloo, ON N2L 3G1, Canada

^b^Faculty of Food Science and Nutrition, Poznań University of Life Sciences, Poznań, Poland

*Corresponding authors:

Janusz Pawliszyn:

Email: janusz@uwaterloo.ca, Tel: +1 519 888 4641

**
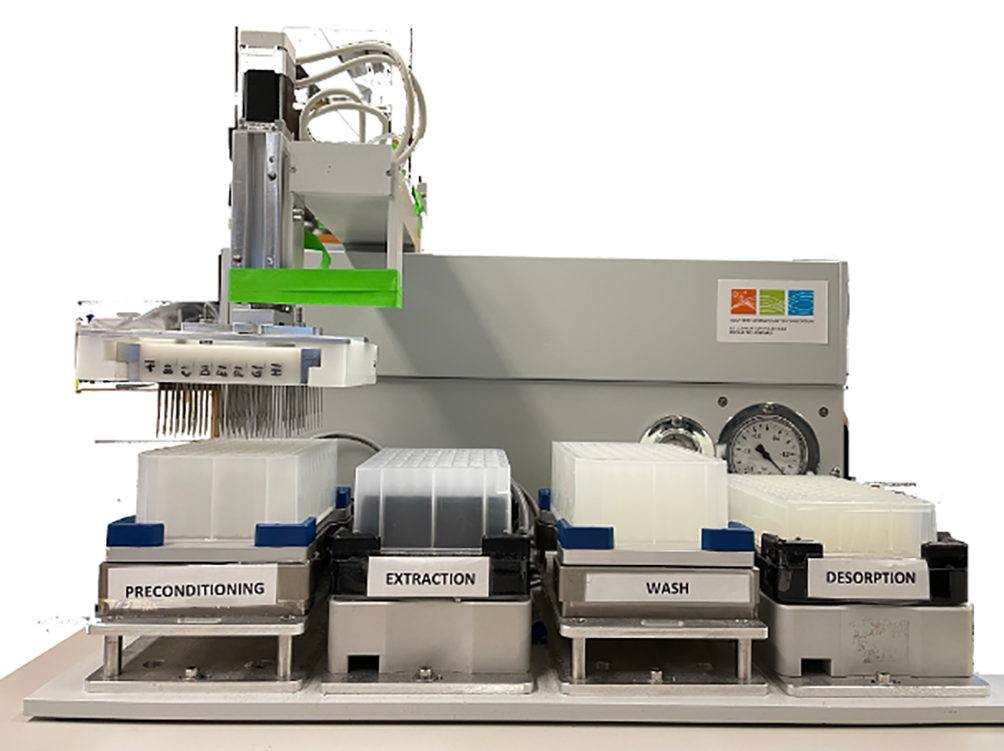
Fig. S1.** The photo of the Concept 96 system.

**
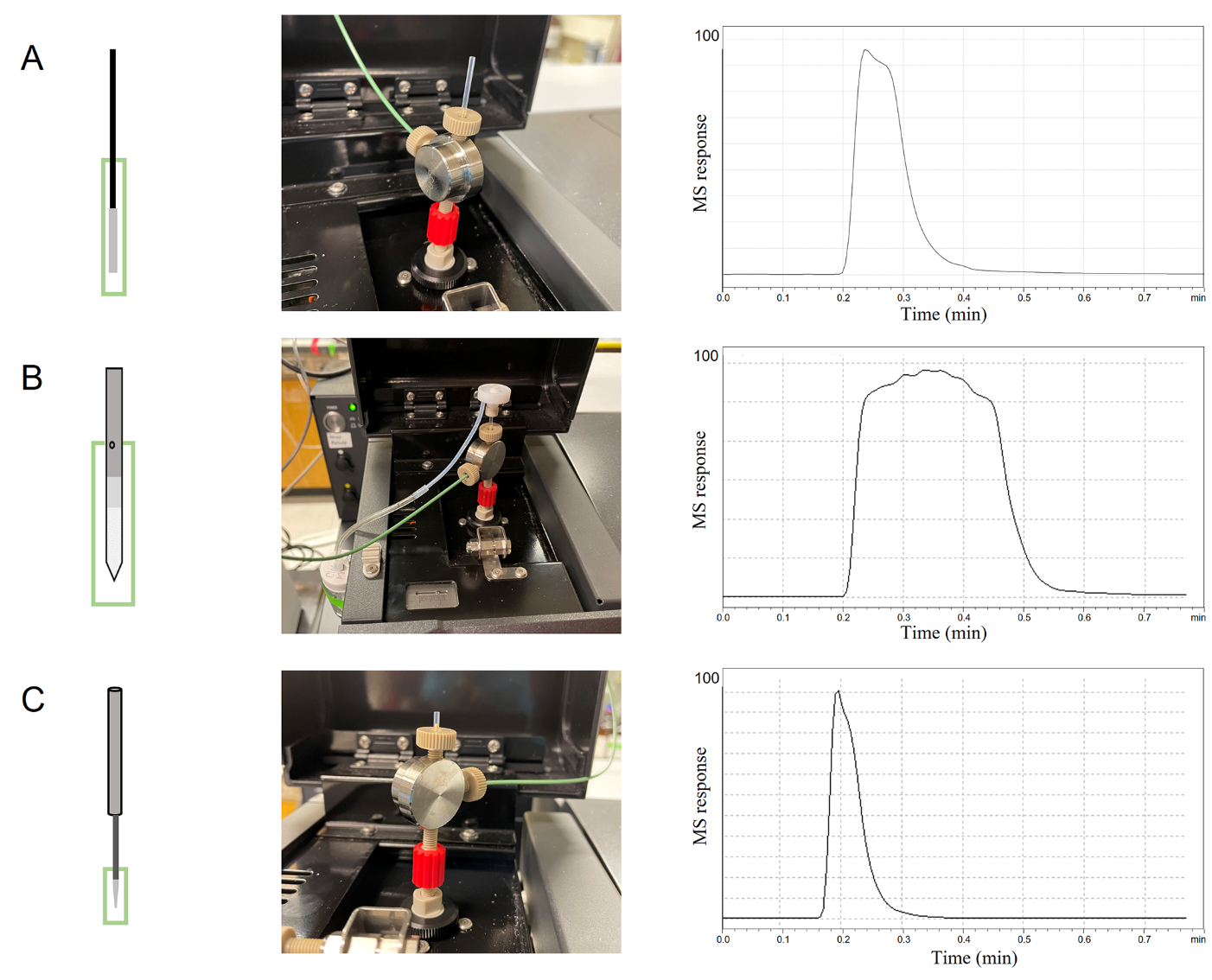
Fig. S2.** The designs and photos of solid-phase microextraction-microfluidic open interface-mass spectrometry (SPME-MOI-MS) interface with different shapes of desorption chambers and related MS spectra. (A) SPME fiber-MOI-MS, (B) SPME blade-MOI-MS, (C) SPME-PESI probe-MOI-MS.

**
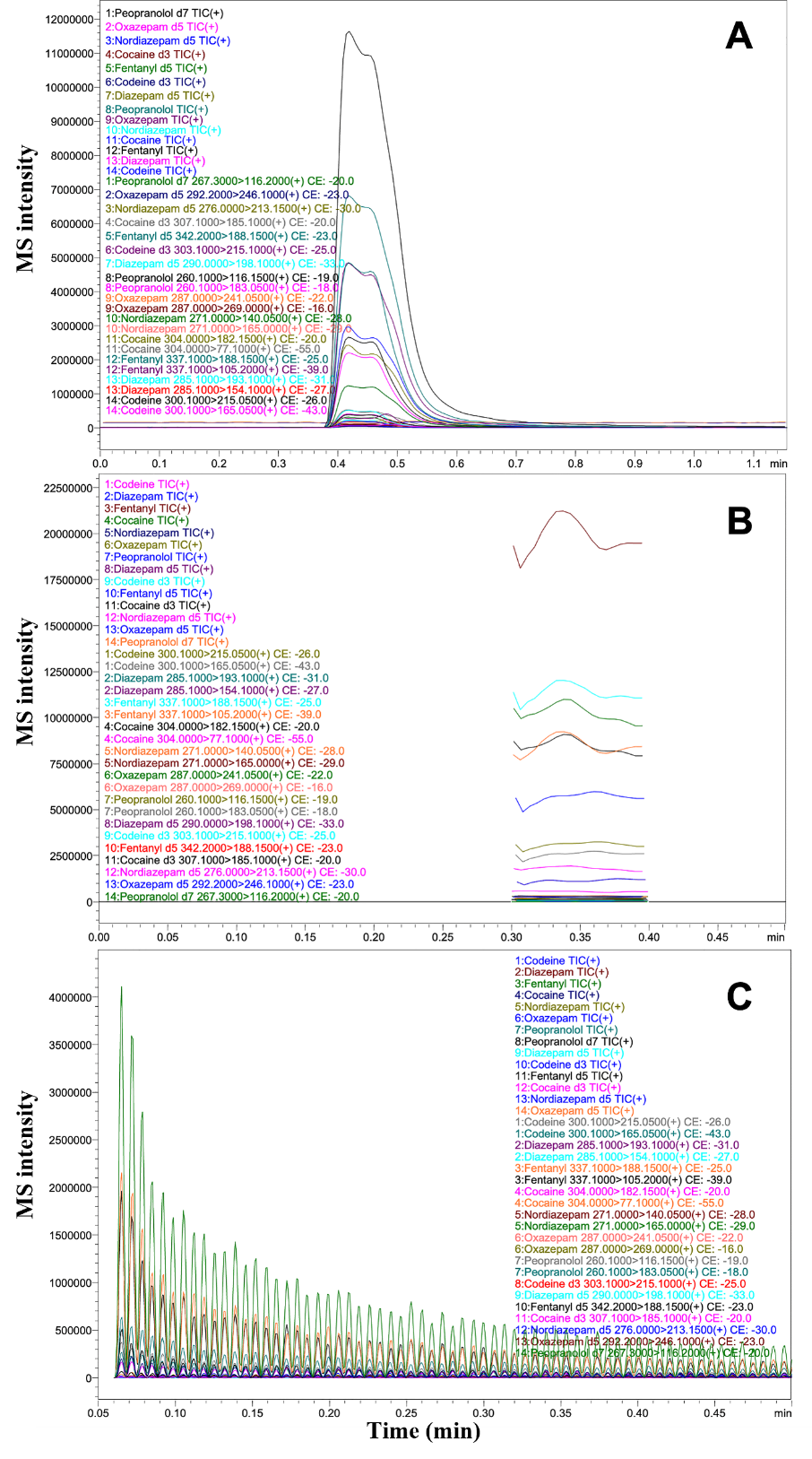
Fig. S3.** The typical MS spectrums of the (A) solid-phase microextraction-microfluidic open interface-mass spectrometry (SPME-MOI-MS), (B) coated blade spray-mass spectrometry (CBS-MS) and (C) solid-phase microextraction-probe electrospray ionization-mass spectrometry (SPME-PESI-MS).

**
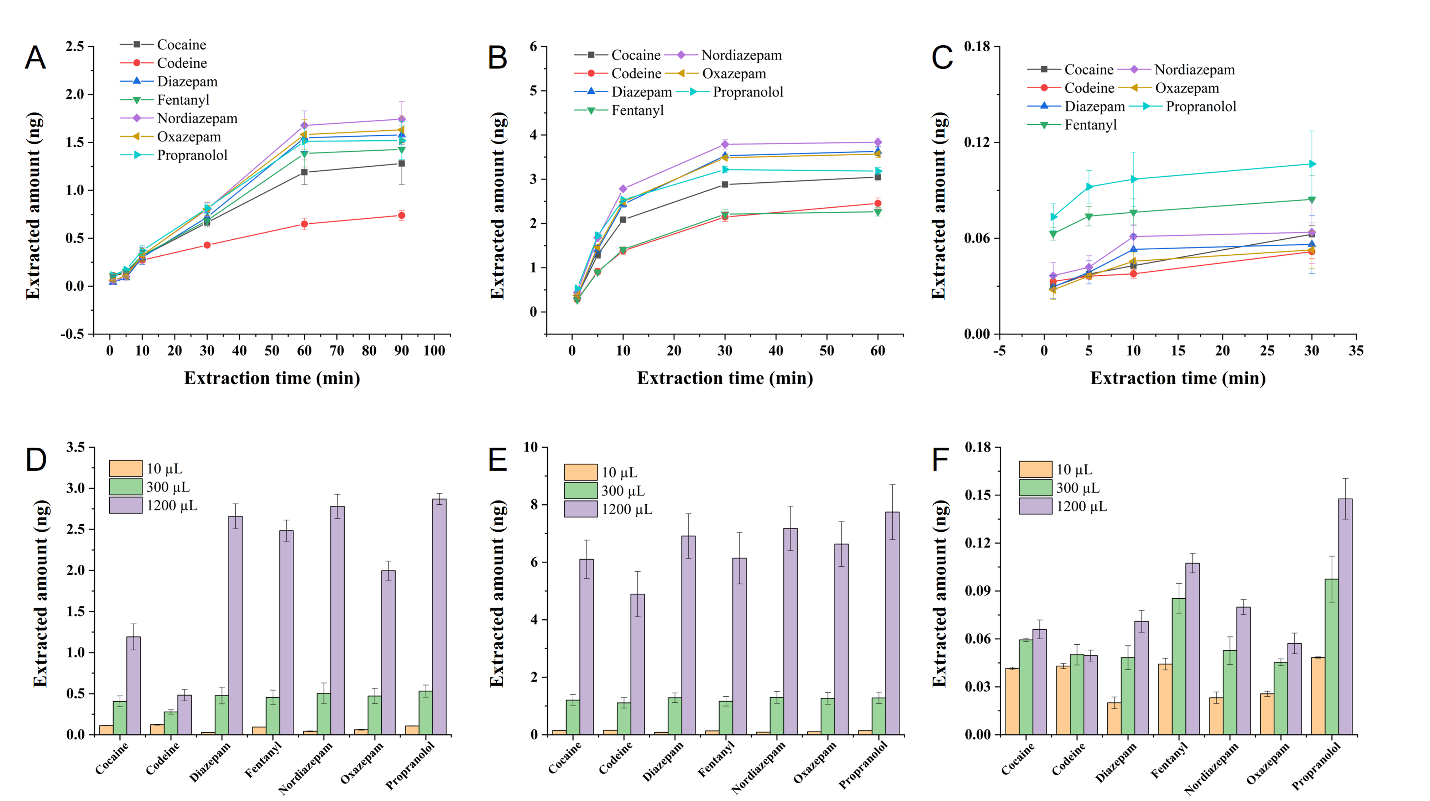
Fig. S4.** The influence of extraction time and sample volume to the extraction performance, (A) and (D): solid-phase microextraction (SPME) fiber; (B) and (E): coated blade spray (CBS) blade; (C) and (F): SPME-probe electrospray ionization (PESI) probe.

**Table S1.** Mass spectrometry parameters of the target compounds. All the columns in Tables need be left aligned. Please check and revise.

| Compounds | Internal Standard | Log P | Precursor Ion (*m/z*) | Product Ion (*m/z*) | Collision energy (eV) | Radio frequency lens (V) |
| --- | --- | --- | --- | --- | --- | --- |
| Cocaine | Cocaine d_3_ | 2.0 | 304.24 | 182.3 | 21.64 | 61 |
|  |  |  |  |  |  |  |
| Codeine | Codeine d_3_ | 1.2 | 300.25 | 165 | 42 | 75 |
|  |  |  |  |  |  |  |
| Fentanyl | Fentanyl d_5_ | 4.1 | 337.3 | 188.08 | 23.41 | 69 |
|  |  |  |  |  |  |  |
| Nordiazepam | Nordiazepam d_5_ | 2.9 | 271.2 | 140.07 | 28.21 | 58 |
|  |  |  |  |  |  |  |
| Oxazepam | Oxazepam d_5_ | 2.2 | 287.2 | 241.05 | 23.24 | 57 |
|  |  |  |  |  |  |  |
| Propranolol | Propranolol d_7_ | 3.0 | 260.29 | 116.13 | 18.69 | 60 |
|  |  |  |  |  |  |  |
| Diazepam | Diazepam d_5_ | 2.6 | 285.2 | 193 | 33 | 81 |

**Table S2.** Lack-of-fit test for the linearities of drugs of abuse analysis in plasma using different solid-phase microextraction (SPME)-mass spectrometry (MS) direct coupling methods.

| Compounds | SPME fiber-MOI-MS | | CBS blade-MOI-MS | | SPME-PESI probe-MOI-MS | | CBS-MS | |
| --- | --- | --- | --- | --- | --- | --- | --- | --- |
|  | F (test) | F (critical) | F (test) | F (critical) | F (test) | F (critical) | F (test) | F (critical) |
| Codeine | 0.02 | 3.89 | 0.29 | 3.89 | /^a^ | / | 0.07 | 3.89 |
| Diazepam | 0.06 | 3.89 | 0.53 | 3.29 | / | / | 0.21 | 3.89 |
| Fentanyl | 0.07 | 2.51 | 2.42 | 2.69 | 0.11 | 2.68 | 0.30 | 2.51 |
| Cocaine | 0.03 | 2.69 | 2.01 | 2.69 | / | / | 0.85 | 2.68 |
| Nordiazepam | 0.04 | 3.29 | 1.14 | 2.93 | 0.03 | 3.89 | 0.08 | 3.29 |
| Oxazepam | 4.79^b^ | 3.89 | 0.07 | 3.89 | / | / | 0.07 | 3.89 |
| Propranolol | 0.04 | 2.69 | 0.30 | 2.77 | 0.02 | 2.93 | 0.08 | 2.51 |

^a^ Calibration curve no data available; MOI: microfluidic open interface; CBS: coated blade spray; PESI: probe electrospray ionization; ^b^ F (test) $>$F (critical).

**Table S3.** Lack-of-fit test for the linearities of drugs of abuse analysis in plasma using solid-phase microextraction-probe electrospray ionization-mass spectrometry (SPME-PESI-MS) and SPME-liquid chromatography-mass spectrometry (SPME-LC-MS) with different devices.

| Compounds | SPME-PESI-MS | | SPME fiber-LC-MS | | CBS blade-LC-MS | | SPME-PESI probe-LC-MS | |
| --- | --- | --- | --- | --- | --- | --- | --- | --- |
|  | F (test) | F (critical) | F (test) | F (critical) | F (test) | F (critical) | F (test) | F (critical) |
| Codeine | 0.00 | 3.89 | 0.24 | 3.89 | 1.17 | 3.29 | / | / |
| Diazepam | /^a^ | / | 0.56 | 3.89 | 0.81 | 3.29 | / | / |
| Fentanyl | 0.00 | 2.51 | 1.01 | 2.68 | 2.32 | 2.51 | 0.01 | 3.89 |
| Cocaine | / | / | 0.71 | 3.29 | 0.73 | 2.68 | / | / |
| Nordiazepam | 0.02 | 3.89 | 0.07 | 3.89 | 0.43 | 3.29 | / | / |
| Oxazepam | / | / | 0.48 | 3.89 | 0.48 | 3.29 | / | / |
| Propranolol | 0.00 | 3.29 | 0.10 | 3.29 | 1.60 | 2.93 | 0.03 | 3.89 |

^a^ Calibration curve no data available; CBS: coated blade spray; PESI: probe electrospray ionization

**Table S4.** Validation data of drugs of abuse analysis in plasma using solid-phase microextraction-microfluidic open interface-mass spectrometry (SPME-MOI-MS) with three different devices.

| Compound | **SPME fiber-MOI-MS** | | | | | | **CBS blade-MOI-MS** | | | | | | **SPME PESI probe-MOI-MS** | | | | | |
| --- | --- | --- | --- | --- | --- | --- | --- | --- | --- | --- | --- | --- | --- | --- | --- | --- | --- | --- |
|  | Accuracy  (%, *n*=4) | | | Precision  (%, *n*=4) | | | Accuracy  (%, *n*=4) | | | Precision  (%, *n*=4) | | | Accuracy  (%, *n*=4) | | | Precision  (%, *n*=4) | | |
|  | 3^a^ | 30 | 75 | 3 | 30 | 75 | 3 | 30 | 75 | 3 | 30 | 75 | 3 | 30 | 75 | 3 | 30 | 75 |
| Codeine | /^b^ | 92 | 107 | / | 5 | 5 | / | 106 | 103 | / | 5 | 4 | / | / | / | / | / | / |
| Diazepam | / | 97 | 105 | / | 3 | 6 | 118 | 103 | 104 | 7 | 9 | 2 | / | / | / | / | / | / |
| Fentanyl | 101 | 102 | 102 | 2 | 2 | 4 | 90 | 110 | 104 | 0 | 0 | 1 | 82 | 95 | 99 | 3 | 10 | 1 |
| Cocaine | 99 | 97 | 107 | 5 | 1 | 3 | 98 | 105 | 102 | 2 | 2 | 1 | / | / | / | / | / | / |
| Nordiazepam | 104 | 97 | 105 | 7 | 5 | 2 | 93 | 100 | 101 | 2 | 3 | 1 | / | 98 | 94 | / | 6 | 10 |
| Oxazepam | / | 108 | 110 | / | 11 | 5 | / | 104 | 106 | / | 4 | 3 | / | / | / | / | / | / |
| Propranolol | 98 | 96 | 107 | 1 | 3 | 1 | 91 | 100 | 101 | 2 | 1 | 1 | 83 | 99 | 104 | 6 | 6 | 4 |

^a^Unit: ng/mL; ^b^No data; MOI: microfluidic open interface; CBS: coated blade spray; PESI: probe electrospray ionization.

**Table S5.** Validation data of drugs of abuse analysis in plasma using three different solid-phase microextraction-mass spectrometry (SPME-MS) methods.

| Compound | SPME fiber-MOI-MS | | | | | | CBS-MS | | | | | | SPME-PESI-MS | | | | | |
| --- | --- | --- | --- | --- | --- | --- | --- | --- | --- | --- | --- | --- | --- | --- | --- | --- | --- | --- |
|  | Accuracy  (%, *n*=4) | | | Precision  (%, *n*=4) | | | Accuracy  (%, *n*=4) | | | Precision  (%, *n*=4) | | | Accuracy  (%, *n*=4) | | | Precision  (%, *n*=4) | | |
|  | 3^a^ | 30 | 75 | 3 | 30 | 75 | 3 | 30 | 75 | 3 | 30 | 75 | 3 | 30 | 75 | 3 | 30 | 75 |
| Codeine | /^b^ | 92 | 107 | / | 5 | 5 | / | 102 | 92 | / | 11 | 10 | / | 100 | 103 | / | 10 | 9 |
| Diazepam | / | 97 | 105 | / | 3 | 6 | / | 86 | 114 | / | 14 | 7 | / | / | / | / | / | / |
| Fentanyl | 101 | 102 | 102 | 2 | 2 | 4 | 89 | 103 | 101 | 2 | 2 | 3 | 104 | 98 | 103 | 5 | 1 | 3 |
| Cocaine | 99 | 97 | 107 | 5 | 1 | 3 | 90 | 102 | 104 | 4 | 4 | 3 | / | / | / | / | / | / |
| Nordiazepam | 104 | 97 | 105 | 7 | 5 | 2 | 110 | 95 | 100 | 8 | 3 | 9 | / | 97 | 101 | / | 13 | 7 |
| Oxazepam | / | 108 | 110 | / | 11 | 5 | / | 98 | 105 | / | 5 | 11 | / | / | / | / | / | / |
| Propranolol | 98 | 96 | 107 | 1 | 3 | 1 | 103 | 98 | 103 | 1 | 3 | 4 | 118 | 94 | 96 | 6 | 4 | 1 |

^a^Unit: ng/mL; ^b^No data; MOI: microfluidic open interface; CBS: coated blade spray; PESI: probe electrospray ionization.

**Table S6.** Validation data of drugs of abuse analysis in plasma using solid-phase microextraction-liquid chromatography-mass spectrometry (SPME-LC-MS) with three different devices.

| Compound | SPME fiber-LC-MS | | | | | | CBS blade-LC-MS | | | | | | SPME-PESI probe-LC-MS | | | | | |
| --- | --- | --- | --- | --- | --- | --- | --- | --- | --- | --- | --- | --- | --- | --- | --- | --- | --- | --- |
|  | Accuracy  (%, *n*=4) | | | Precision  (%, *n*=4) | | | Accuracy  (%, *n*=4) | | | Precision  (%, *n*=4) | | | Accuracy  (%, *n*=4) | | | Precision  (%, *n*=4) | | |
|  | 3^a^ | 30 | 75 | 3 | 30 | 75 | 3 | 30 | 75 | 3 | 30 | 75 | 3 | 30 | 75 | 3 | 30 | 75 |
| Codeine | /^b^ | 101 | 107 | / | 4 | 3 | 114 | 97 | 101 | 6 | 2 | 2 | / | / | / | / | / | / |
| Diazepam | / | 104 | 112 | / | 2 | 4 | / | 97 | 99 | / | 4 | 1 | / | / | / | / | / | / |
| Fentanyl | 85 | 104 | 109 | 1 | 1 | 1 | 105 | 96 | 100 | 0 | 0 | 0 | / | 102 | 106 | / | 4 | 3 |
| Cocaine | 81 | 100 | 107 | 9 | 3 | 2 | 97 | 99 | 101 | 2 | 2 | 1 | / | / | / | / | / | / |
| Nordiazepam | / | 96 | 109 | / | 3 | 3 | 114 | 98 | 102 | 4 | 2 | 1 | / | / | / | / | / | / |
| Oxazepam | / | 102 | 109 | / | 2 | 3 | 112 | 99 | 99 | 1 | 2 | 1 | / | / | / | / | / | / |
| Propranolol | 88 | 103 | 108 | 1 | 1 | 2 | 111 | 97 | 101 | 2 | 1 | 2 | 88 | 103 | 108 | 1 | 1 | 1 |

^a^Unit: ng/mL; ^b^No data; CBS: coated blade spray; PESI: probe electrospray ionization.
